# Advancements in artificial intelligence for ultrasound diagnosis of ovarian cancer: a comprehensive review

**DOI:** 10.3389/fonc.2025.1581157

**Published:** 2025-06-12

**Authors:** Chenxin Tang, Zhenbin Xu, Hongpeng Duan, Shengmin Zhang

**Affiliations:** ^1^ Health Science Center, Ningbo University, Ningbo, China; ^2^ Department of Ultrasound Medicine, The First Affiliated Hospital of Ningbo University, Ningbo, China

**Keywords:** artificial intelligence, ultrasound imaging, ovarian cancer, machine learning, deep learning

## Abstract

Ovarian cancer, as a common gynecological malignancy, is often found at an advanced stage clinically. Thus, improving the early diagnosis of ovarian cancer is crucial for the survival rate of patients. Ultrasound examination is the main method for ovarian cancer screening, but it is greatly influenced by the operator’s experience and technique, increasing the risk of misdiagnosis and missed diagnosis. Artificial intelligence uses computers to learn from input data and has already made significant progress in image recognition. Applying artificial intelligence to ultrasound diagnosis of ovarian cancer can enhance diagnostic accuracy, providing earlier treatment for patients. This article reviews the current application of artificial intelligence in the ultrasound diagnosis of ovarian cancer, in order to provide a reference for subsequent clinical diagnosis and treatment.

## 1 Introduction

Ovarian cancer is recognized globally as one of the top three gynecological malignancies, with mortality rates only exceeded by those of cervical cancer. In 2022, approximately 324,398 new cases and 206,839 deaths were recorded (1). Early detection and diagnosis of ovarian cancer can slow down the progression of the disease and improve the survival rate of patients. The classification of ovarian cancer is primarily based on its origins, with epithelial ovarian cancer being the most prevalent, accounting for over 95% of cases. High-grade plasmacytoid ovarian cancer is the most common histological subtype of epithelial ovarian cancer, often presenting with extensive pelvic and abdominal metastases at an early stage. Genetic analysis indicates that 96% of High-grade plasmacytoid ovarian cancer cases exhibit mutations in the TP53 gene, leading to a loss of protein function and the inability of cells to effectively repair DNA, thereby promoting cancer cell growth and resulting in a poor prognosis (2–4). Consequently, accurate staging of ovarian cancer is imperative for the selection of subsequent treatment options.

The prognosis of ovarian cancer patients is contingent upon the stage of the disease at the time of diagnosis, in addition to histologic typing. The five-year survival rate for patients diagnosed at an early stage can exceed 90%, while the survival rate for patients diagnosed at a late stage plummets to less than 30% (5). Ovarian cancer lacks clear clinical symptoms and specific screening methods, which often leads to delayed diagnosis. Consequently, there is an urgent need to improve early diagnosis to enhance clinical outcomes for patients (6, 7). The gold standard for diagnosing ovarian cancer remains pathological examination. However, before obtaining tissue samples through surgery or biopsy, clinicians typically begin with a physical examination and then use imaging techniques and tumor marker tests to evaluate the likelihood of malignancy. The most commonly used biomarker for ovarian cancer, CA125, has limited diagnostic specificity as elevated levels may also occur in benign conditions such as endometriosis. Consequently, CA125 should be interpreted as an auxiliary indicator rather than a definitive diagnostic tool, and its clinical utility depends on integration with imaging findings for comprehensive assessment. Among imaging modalities, ultrasound, computed tomography (CT), magnetic resonance imaging (MRI), and positron emission tomography (PET) are commonly used to assess ovarian masses. CT and MRI are particularly valuable for preoperative staging, providing detailed cross-sectional views of anatomical structures, lymph node involvement, and tumor spread (8, 9). However, CT exposes patients to radiation, while MRI is time-consuming, expensive, and has certain contraindications. As a result, MRI is generally reserved as a supplementary tool when ultrasound yields inconclusive results. PET, though useful for detecting distant metastases and lesions smaller than 5 mm (10, 11), is limited by its high cost, radiation exposure, and relatively low spatial resolution, making it unsuitable for routine screening.

**Figure 1:**
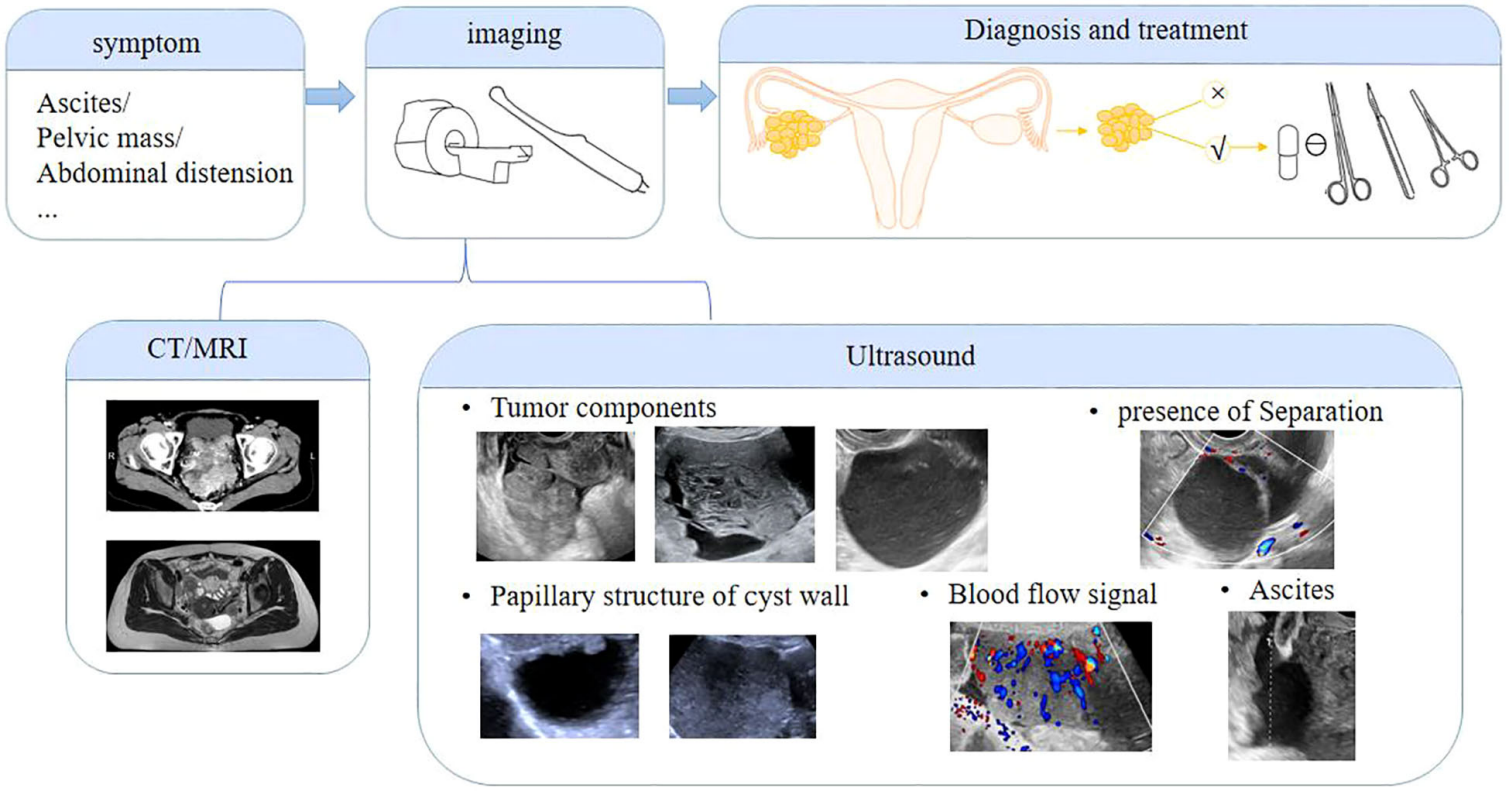
The following schematic diagram illustrates the ultrasound evaluation of a pelvic mass. In the context of pelvic masses, ultrasound can be utilized for the following purposes: assessment of size, composition, segregation, cystic wall nodules, blood flow signal, and the presence of ascites.

Ultrasound, especially transvaginal sonography (TVS), is a non-invasive, cost-effective, and widely accessible imaging method that provides high-resolution, real-time dynamic imaging of ovarian masses. Unlike static imaging techniques such as CT and MRI, ultrasound allows clinicians to assess tissue mobility and vascular patterns, which can be particularly useful in detecting malignancies associated with endometriosis (9). TVS evaluates key features such as mass size, morphology, and blood flow while identifying potential signs of malignancy, including papillary projections, thick septations, and ascites (**Figure 1**). Additionally, serial ultrasound examinations can help monitor disease progression over time (12, 13). Despite its advantages, ultrasound has notable limitations. Diagnostic accuracy heavily depends on the operator’s skill and experience, and image quality may be affected by factors such as the patient’s age or prior pelvic surgery. False-positive or false-negative results can lead to unnecessary anxiety or delayed diagnosis (9, 13, 14). While ultrasound remains a first-line diagnostic tool due to its safety and accessibility, further standardization is needed to improve its reliability in early ovarian cancer detection.

To reduce subjectivity in ultrasound interpretation, standardized scoring systems such as the International Ovarian Tumor Analysis (IOTA) model and the Risk of Malignancy Index (RMI) have been developed. These systems integrate clinical variables (e.g., age, CA125 levels) with ultrasound findings (e.g., mass diameter, solid components) to enhance diagnostic accuracy (15, 16). Recent advancements in artificial intelligence (AI) have further refined ultrasound diagnostics. Machine learning (ML) and deep learning (DL) techniques—particularly convolutional neural networks (CNNs)—have shown remarkable success in medical image analysis (17, 18). AI-assisted ultrasound has achieved sensitivity and specificity rates of 91% and 92%, respectively, in distinguishing benign from malignant tumors (19), enabling earlier and more precise clinical decision-making. AI also serves as a valuable aid for less experienced sonographers, helping reduce diagnostic variability (20, 21). When combined with tumor markers such as CA-125, AI models improve malignancy risk assessment (22), marking a significant step forward in the early detection and management of ovarian cancer.

This comprehensive review presents recent advances in the application of artificial intelligence to ultrasound examination of ovarian cancer, including benign and malignant classification, adjuvant diagnosis, pathological staging, and prognostic applications. We systematically searched PubMed and Google Scholar for relevant studies published between January 1999 and December 2024 using the following specific keywords: “artificial intelligence”, “computer-aided”, “neural networks”, “machine learning”, “deep learning”; “ultrasound”, “ultrasound image”, “transvaginal”, “transabdominal”; “ovarian”, “ovarian tumor”, “ovarian cancer”, “epithelial ovarian cancer”. The review also summarizes publicly available databases supporting AI development in this field. Finally, we discuss the key challenges in clinical implementation of these AI models and examine current approaches to address these limitations. The findings provide valuable insights for researchers and clinicians working at the intersection of AI and ovarian cancer diagnostics.

## 2 Potential applications of artificial intelligence in early diagnosis of ovarian cancer

### 2.1 Artificial intelligence-based method for benign and malignant ovarian cancer identification

The differentiation of benign and malignant ovarian cancer needs to combine various factors such as clinical manifestations, imaging manifestations and laboratory examinations. Currently, histopathology remains the gold standard for determining the benign or malignant nature of a mass. However, with the rapid development of AI technology, especially in the field of ultrasound imaging and deep learning, AI models are gradually becoming an emerging tool for early diagnosis of ovarian cancer. In recent years, researchers have developed a variety of AI models to assist ultrasound diagnosis by extracting imaging histology features and quantifying mass characteristics, thereby improving the identification of benign and malignant masses. The application of artificial intelligence in ovarian cancer ultrasound diagnosis has evolved significantly since its inception. The field’s foundational work dates back to 1999 when initial AI models demonstrated potential for predicting ovarian malignancy risk (23). A pivotal advancement occurred in 2010 with Lucidame et al.’s development of the OVHS system, which achieved remarkable 98% sensitivity in subsequent validation studies (24). Subsequent studies have progressively refined AI’s diagnostic capabilities. Acharya’s team established an ML model with 81.4% sensitivity and 76.3% specificity by analyzing 2D/3D ultrasound images from 469 patients (25). Martinez-Mas et al. subsequently compared four classical ML algorithms (K-Nearest Neighbors (KNN), Linear Discriminant (LD), Support Vector Machine (SVM) and Extreme Learning Machine (ELM)) for ovarian tumor classification, finding that LD, SVM and ELM achieved >85% accuracy versus KNN’s <60% performance (26). These comparative studies provided crucial insights into algorithm selection for optimal diagnostic performance. The advent of deep learning brought further improvements. Wang et al.’s deep convolutional neural network (DCNN) model achieved 75% accuracy in tri-class (benign/borderline/malignant) differentiation, with 89% sensitivity for malignant cases (27). Hsu et al. advanced the field by evaluating 10 CNN models through rigorous cross-validation, ultimately developing an ensemble model with 91% sensitivity and 92% specificity (19). These advances not only demonstrated AI’s growing reliability in ovarian tumor assessment but also paved the way for more sophisticated multimodal approaches. Qi et al. demonstrated enhanced predictive performance by combining ultrasound features with clinical data (age, ascites, CA125), with IDI (Integrated Discrimination Improvement) improvements ≥0.154 for benign/non-benign and ≥0.815 for borderline/malignant differentiation (28). Similarly, Du et al. developed a fusion model integrating CNN analysis with radiomics and clinical features, achieving AUCs(area under the curve) of 0.83-0.85 across tumor types (29). Such multimodal strategies represent a significant leap forward, offering both improved diagnostic precision and the potential to reduce unnecessary invasive procedures. The American College of Radiology’s consensus guidelines established the Ovarian-Adnexal Reporting and Data System (O-RADS), a standardized framework that classifies malignancy risk across six categories (O-RADS 0-5) to guide clinical decision-making (30). The clinical relevance of recent AI advancements is particularly evident in their strong alignment with this established system. Chen et al.’s DL model showed strong concordance with both expert assessment and O-RADS criteria (31), while Christiansen et al. confirmed comparable specificity between DL analysis (n=3,077 images) and expert evaluation (32). These validation studies provide compelling evidence for AI’s readiness for clinical integration in ovarian mass assessment. **Table 1** demonstrates the performance of AI models in the benign and malignant differentiation of ovarian cancer in some studies in recent years, including its key indexes such as sensitivity, specificity and AUC value. These studies have fully demonstrated the importance of AI models in improving the accuracy and efficiency of ultrasound diagnosis.

**Table 1:** Identification of benign and malignant ovarian cancer based on artificial intelligence modeling.

Reference	Design	Number of patients	AI technology	Highlights
Identification of benign and malignant tumors				
Hsu 2022 (19)	Retrospective, single-center	587	DL	The highest of ten single models: accuracy90.51 ± 4.36% sensitivity89.77 ± 4.16% specificity92.00 ± 5.95% The ensemble classifier method: accuracy92.15 ± 2.84% sensitivity91.37 ± 3.60% specificity92.92 ± 4.00%
Tailor 1999 (23)	Retrospective, single-center	67	DL	artificial neural network output 0.45: sensitivity 100% specificity 98.1%
Lucidame 2010 (24)	Prospective, multi-center	264	ML	OVHS: sensitivity98%, specificity88% Combining standard TVS and OVHS improved TVS specificity from 66% to 92%
Acharya 2018 (25)	Retrospective, single-center	469	ML	accuracy 80.60 ± 0.5%, sensitivity 81.40%, specificity 76.30%
Martinez-Mas 2019 (26)	Retrospective, single-center	Unspecified	ML	LD, SVM and ELM: more than 85% of accuracy; KNN: less than 60% of accuracy
Wang 2021 (27)	Retrospective, single-center	265	DL,	The best DL model in the testing dataset: classify benign and non-benign tumors, AUC 0.96, sensitivity 0.91, specificity 0.91. borderline vs malignant: AUC 0.91, sensitivity 0.98, specificity 0.74. directly classifying the three categories: accuracy 0.75
Qi 2021 (28)	Retrospective, single-center	279 (10-fold cross-validation)	ML radiomics	combined clinical-radiomics (CCR) model: classify benign and non-benign: AUC 0.937, borderline vs malignant: AUC 0.924
Du 2024 (29)	Retrospective, single-center	849 (training 680; testing169)	DL, radiomics	DL radiomics (DLR)-clinical model: testing AUC 0.90 (micro-average),0.84(macro-average)
Chen 2022 (31)	Retrospective, single-center	422 (training 296; validation 41; testing85)	DL	DL-_feature_: AUC 0.93, sensitivity 92.0%, specificity 85%. DL-_decision_: AUC 0.90, sensitivity 92.0%, specificity 80%
Christiansen 2021 (32)	Retrospective, multi-center	758 (training 508; validation 100; testing150)	DL	Model 1: sensitivity 96.0%, specificity 86.7%. Model 2: sensitivity 97.1%, specificity 93.7%

### 2.2 The role of artificial intelligence in improving diagnostic consistency of ultrasonographers

Although ultrasound, as a common tool for ovarian tumor diagnosis, is widely used in clinical practice, the accuracy of its diagnosis is highly correlated with physicians’ experience (33). To address this issue, the introduction of AI models has significantly improved the consistency and accuracy of sonographers in ovarian cancer diagnosis. For example, the ADNEX model and the Simple Rule (SR) model developed by the IOTA group provide standardized tools for ultrasound diagnosis. A comparison of the accuracy of these models with that of expert assessment revealed comparable results, with the former greatly reducing subjective errors in diagnosis (34, 35). The diagnostic accuracy of the IOTA-ADNEX model in ovarian tumors was also demonstrated in a study by Soo Young Jeong et al. (36) Additionally, scholars have compared SR with the SRU consensus guidelines and similarly demonstrated the diagnostic ability of SR with an AUC of up to 0.98 for diagnosing malignant tumors (37). The study also compared the accuracy of mass classification using four models by grouping junior doctors with senior doctors and showed that all four models were able to compensate for the shortcomings of junior doctors in predicting the risk of malignancy (38).

The application of computer-aided diagnosis (CAD) technology in ultrasound image analysis further compensates for the shortcomings of junior doctors in diagnosis (39). This is well demonstrated in the study by Gao et al., who developed a DCNN model for automated ultrasound image evaluation. Their results revealed significant improvements in diagnostic performance among junior sonographers when assisted by the DCNN, with sensitivity increasing from 70.4% to 82.7% and specificity rising from 80.1% to 88.7% (40). Subsequently, Xiang et al. created OvcaFinder, an advanced model integrating deep learning analysis of ultrasound images with O-RADS classification and clinical data. This integrated system demonstrated superior performance in both internal and external validation while significantly reducing false-positive rates and improving diagnostic consistency (**Table 2**) (41). Further advancing the field, Chiappa’s team developed a novel AI model that minimizes dependence on physician experience. Their system enables accurate tumor characterization (benign vs malignant) through simple identification of mass composition (solid, cystic, or heterogeneous), achieving impressive AUC values of 0.87,0.88,0.89 (42). These developments collectively highlight the transformative potential of CAD systems in enhancing diagnostic accuracy and standardizing ultrasound interpretation across varying levels of clinical experience.

**Table 2:** Artificial intelligence improves diagnostic consistency for sonographers.

Reference	Design	Number of patients	AI technology	Highlights
Improving diagnostic consistency for sonographers				
Gao 2022 (40)	Retrospective, multi-center	107624 (training 105532; internal validation868; Two external validations:335,889)	DL	AUC:0.911 (internal validation), 0.870 (external validation-1), 0.831 (external validation-2) After auxiliary diagnosis: accuracy increased by 9.3%, sensitivity increased by 12.3%
Xiang 2024 (41)	Retrospective, single-center	1111 (training 532; validation 63; internal validation129; external validation:387)	DL	OvcaFinder: AUC:0.978 (internal validation), 0.947 (external validation); After auxiliary diagnosis: the false positive rate decreased by 13.4% in the internal validation and 8.3% in the external validation
Chiappa 2021 (42)	Retrospective, single-center	241 (Solid 95, Cystic 66, Motley 80.)	ML	Solid: accuracy80%, sensitivity 78%, specificity 83%, AUC 87%. Cystic: accuracy 87%, sensitivity 75%, specificity 90%, AUC 88%. Mixed: accuracy 81%, sensitivity 81%, specificity 81%, AUC 89%

### 2.3 Pathologic staging of ovarian cancer and prediction of prognosis

Epithelial ovarian cancer, the most common type of ovarian cancer, can be classified according to WHO as type I or type II. Type I tumors usually appear inert and are mostly confined to the ovary at the time of diagnosis. In contrast, type II tumors appear highly aggressive and progress rapidly (43). Therefore, distinguishing the histologic type preoperatively is crucial for the choice of treatment strategy and prognosis of patients. Addressing this clinical need, Tang et al. conducted a comprehensive retrospective analysis of ultrasound images from 154 epithelial ovarian cancer patients. Through rigorous feature selection, they identified seven optimal imaging characteristics and developed a robust predictive model capable of accurately differentiating between type I and type II. The model showed good performance in the training set and test set, with AUCs of 0.817 and 0.731, respectively. in addition, they combined clinical indicators with images to construct a predictive model and obtained higher AUCs (**Table 3**) (44). The results of this study suggest that the type of pathology does not necessarily have to be performed by invasive examination, but can also be predicted by ultrasound images.

**Table 3:** AI based ultrasound pathological classification and prognosis prediction of ovarian cancer.

Reference	Design	Number of patients	AI technology	Highlights
Pathological classification				
Tang 2022 (44)	Retrospective, single-center	154 (training 93; testing 61)	ML radiomics	Radiomics model: AUC 0.817 (training), 0.731 (testing); Comprehensive model: AUC 0.982 (training), 0.886 (testing)
Prediction of survival outcome				
Arezzo 2022 (46)	Retrospective, single-center	64 (five-fold cross- validation)	ML	The best ML model predict 12month PFS: accuracy 93.7%, AUROC 0.92
Zuo 2024 (47)	Retrospective, multi-center	514 (training 386; internal validation:76; external validation 52)	DL	predictive overall survival (OS) and recurrence-free survival (RFS): concordance indices ranging from 0.773 to 0.794.
Qi 2024 (48)	Retrospective, multi-center	401 (training332; testing 79)	ML radiomics	predict lymph node metastasis: Radiomics model: AUC 0.899 (training), 0.855 (testing); Radiomics-clinical model: AUC 0.930 (training), 0.881 (testing);

The overall survival rate of ovarian cancer at the time of diagnosis is approximately 40%, and factors such as the stage of the disease at the time of diagnosis, histologic classification, and age can all influence the prognosis of the patient (45). Prediction of a patient’s prognosis can prolong survival by selecting a personalized treatment plan and follow-up time for the patient. In this regard, Arezzo et al. retrospectively analyzed the ultrasound images and clinical features of 64 patients with epithelial ovarian cancer and used three different ML algorithms (Logistic Regression (LR), Random Forest (RFF) and KNN) to predict 12-month PFS(progression-free survival), and ultimately found that the RFF had the best performance, achieving an area under receiver operating characteristic curve (AUROC) of 0.92. This helps to stratify the patients and provide better treatment strategies (46). Similarly, models have been used to predict the recurrence rate of patients. a study by Zuo et al. validated the ability of models to predict OS (overall survival) and RFS (recurrence-free survival) in Ovarian cancer patients. A radiomics prognostic model based on ultrasound images was developed by extracting personalized statistical radiomics features from preoperative ultrasound images of 514 ovarian cancer patients. The results showed a concordance index range of 0.773-0.794 for OS and RFS (47). This provides a basis for individualized prognostic assessment of patients. In addition, the status of lymph nodes is a key factor affecting prognosis. Qi et al. constructed a combined model by extracting relevant features from preoperative ultrasound images and clinical information to predict the lymph node status of patients with high-grade plasmacytoid ovarian cancer. The results showed that the AUC of this method in the training set and test set were 0.930 and 0.881, respectively (48). This method is noninvasive and practical, which helps to assess the patient’s condition, and choose the appropriate treatment plan.

## 3 Future directions in Ai for ultrasound diagnosis of ovarian cancer

The future of artificial intelligence in the diagnosis of ovarian cancer via ultrasound holds immense promise but also faces key challenges. Based on current progress and limitations, three critical areas are highlighted for future research and development:

### 3.1 Integration of multimodal imaging and biological data

merging evidence demonstrates that multimodal integration approaches significantly outperform single-parameter models in ovarian cancer diagnosis. Current research highlights the superior diagnostic accuracy of models combining clinical parameters with radiomics features, while increasingly incorporating advanced genomic and transcriptomic analyses. Future investigations should focus on synthesizing multiparametric imaging data (CT, MRI, and ultrasound) with tumor-specific biological profiles, including (1): gene expression signatures, (2) pathway activity mapping (particularly metabolic and angiogenic pathways), and (3) molecular marker panels. This multidimensional integration framework will enable more comprehensive tumor characterization and accelerate precision oncology implementation. For example, the fusion of ultrasound and MRI, combined with tumor-specific biological data, can significantly improve early detection rates, enable personalized therapeutic strategies, and address limitations inherent in relying on single-modality imaging. Additionally, incorporating biological markers and pathway-based evaluations can refine patient stratification, leading to more precise diagnostic and treatment protocols tailored to molecular and functional characteristics of individual tumors.

### 3.2 Improving data quality and model interpretability

The scarcity and heterogeneity of high-quality annotated datasets remain major barriers to developing robust AI models. Current studies are predominantly limited by small-scale datasets (typically dozens to hundreds of cases) and heavy reliance on single-center collections, resulting in inadequate diversity of case representation. These fundamental limitations potentially undermine both the reliability of extracted imaging features and the clinical generalizability of the developed models. Future studies should focus on building open-access, standardized image repositories by encouraging inter-institutional data sharing while addressing discrepancies arising from differences in imaging devices, protocols, and post-processing methods. A standardized, publicly accessible dataset enables rigorous external validation of research findings, significantly enhancing their reliability and clinical applicability. In addition, deep learning techniques, such as data augmentation, unsupervised learning, and transfer learning, are expected to play critical roles in improving data quality and reducing reliance on manual annotations. A critical limitation of current research lies in its predominantly retrospective design, with prospective studies remaining scarce, consequently compromising the overall evidence quality. Concurrently, the interpretability of the models is a pivotal research component. The transparency of the models enables clinicians to ascertain the rationale underlying the AI predictions, thereby fostering their confidence in the outcomes of the decisions made.

### 3.3 Addressing ethical and practical implementation challenges

A number of ethical and practical issues must be resolved before AI models can be widely adopted in clinical settings. These issues include the question of how to ensure data privacy and security, the question of who should bear responsibility when AI decisions are incorrect, the question of whether the public will be able to accept the use of AI models for diagnosis, and the question of whether doctors will overly rely on AI at the expense of improving their own expertise. In addition to ethical issues, AI models face several key challenges in real-world clinical applications. The first and foremost issue is the phenomenon of overfitting, whereby models make predictions based solely on the features of the training dataset, which can lead to their poor performance in subsequent validation. This deviation of predictions from reality can produce misleading judgments and severely hinder the translation of AI models to the clinic. Overfitting usually stems from insufficient training data, and thus requires an increase in data complexity during model construction, which can be achieved by using strategies such as pre-trained models on large-scale medical imaging datasets, cross-validation, etc. Another key challenge is the issue of generalization ability of the models. Most current models perform well on training data, but their effectiveness decreases during validation, which reflects the model’s dependence on training data and limits its breadth of clinical applications. The model generalization ability can be enhanced by data augmentation (e.g., rotation, cropping, etc.), integrated learning, migration learning, and federated learning. In addition, the learning data of the model mostly come from common image features, and its recognition and prediction accuracy is often insufficient when encountering complex and rare images. Therefore, the input of rare case features should be consciously increased during model construction, and the data imbalance problem should be solved by methods such as data enhancement and weighted loss function. Although barriers to clinical adoption remain, accumulating evidence substantiates the diagnostic efficacy of AI-assisted ultrasound in ovarian cancer detection.
